# Cervical Myelopathy From Pseudogout: A Rare Case of Calcium Pyrophosphate Deposition

**DOI:** 10.7759/cureus.69829

**Published:** 2024-09-21

**Authors:** Mohammed Khaleel I.KH. Almadhoun, Osamah O Ta'amneh

**Affiliations:** 1 Medicine and Surgery, Mutah University, Karak, JOR; 2 General Practice, National Center for Diabetes, Endocrinology and Genetic Diseases, Irbid, JOR

**Keywords:** calcium pyrophosphate, chronic neck pain, compression cervical myelopathy, myeloradiculopathy, pathology of pseudogout

## Abstract

Calcium pyrophosphate dihydrate deposition (CPPD), or pseudogout, typically affects joints like the knee and shoulder but can also deposit in spinal structures, sometimes leading to myeloradiculopathy with severe neck pain and upper limb weakness. Mild cases are managed with anti-inflammatory drugs, while severe cases require surgical decompression. We report a rare case of pseudogout causing cervical spine myelopathy at the C1-2 level, discovered during spine surgery and confirmed by pathology. After removing the deposits, the patient showed significant improvement, emphasizing the need to consider pseudogout in cases of acute neck pain with neurological symptoms.

## Introduction

Calcium pyrophosphate dihydrate (CPPD) deposition disease, commonly referred to as pseudogout, is a crystal-induced arthropathy that primarily targets larger, weight-bearing joints such as the knees, hips, and shoulders. It is characterized histologically by weakly birefringent rhomboid crystals, in contrast to the needle-shaped crystals observed in gout​ [1]. CPPD can present with a wide spectrum of clinical manifestations, ranging from asymptomatic chondrocalcinosis to acute inflammatory arthritis, often mimicking conditions such as gout or rheumatoid arthritis [2]. Risk factors for CPPD include aging, osteoarthritis, metabolic disorders, and hereditary factors, with conditions like hyperparathyroidism, hypomagnesemia, and hypophosphatasia also being implicated [1,2].

Although CPPD most commonly affects peripheral joints, involvement of the spine is rare but significant. When CPPD deposits in the cervical spine, it can lead to calcification of the ligamentum flavum, potentially causing spinal cord compression and myelopathy. The underlying mechanisms behind spinal CPPD deposition remain unclear, though metabolic dysfunctions, genetic predisposition, and sporadic occurrences have been suggested [2].

In this case report, we present an unusual instance of cervical spine myelopathy caused by CPPD deposition in the ligamentum flavum, which was discovered incidentally during posterior cervical decompression and fusion surgery. This case underscores the importance of considering pseudogout in the differential diagnosis of cervical myelopathy, particularly in older patients with risk factors for CPPD deposition [1,2].

## Case presentation

A female patient in her early 60s with a medical history of diabetes, chronic kidney disease, rheumatoid arthritis, anxiety, and peripheral vascular disease presented with a three-month history of increasing neck stiffness, difficulty with coordination, and left upper extremity numbness and weakness. The patient also reported episodes of dizziness and occasional unsteadiness while walking.

Physical examination revealed limited range of motion in the cervical spine, tenderness along the left side of the neck, and a positive Spurling's test. Neurological examination indicated weakness, hyperreflexia, and reduced sensation in the left upper extremity. There was also bilateral lower extremity hyperreflexia and sensory deficits, alongside an ataxic gait.

**Figure 1:**
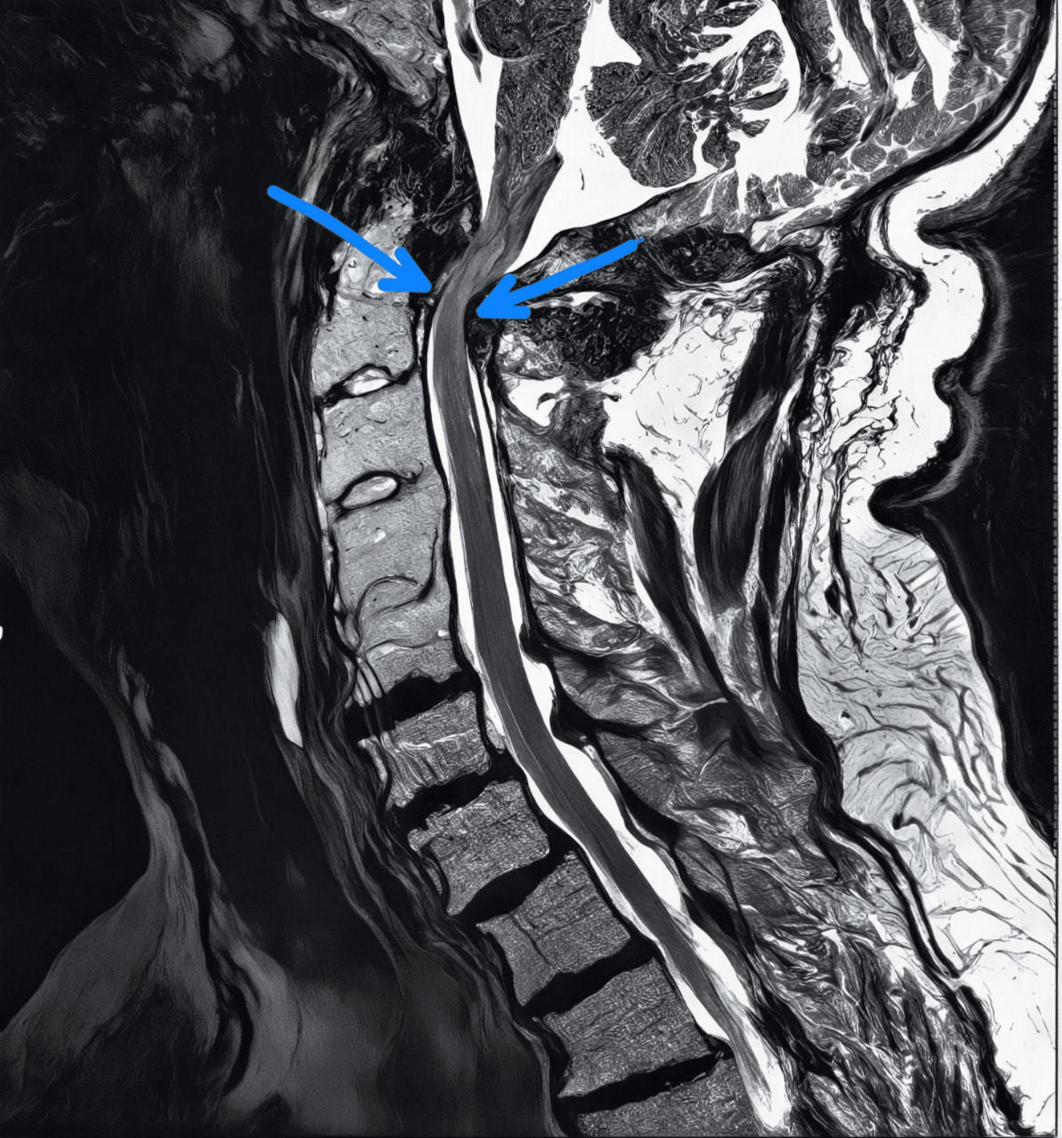
Cervical spine MRI Sagittal T2-weighted MRI of the cervical spine highlighting significant stenosis at the C1-2 junction, marked by arrows indicating both anterior and posterior compression.

**Figure 2:**
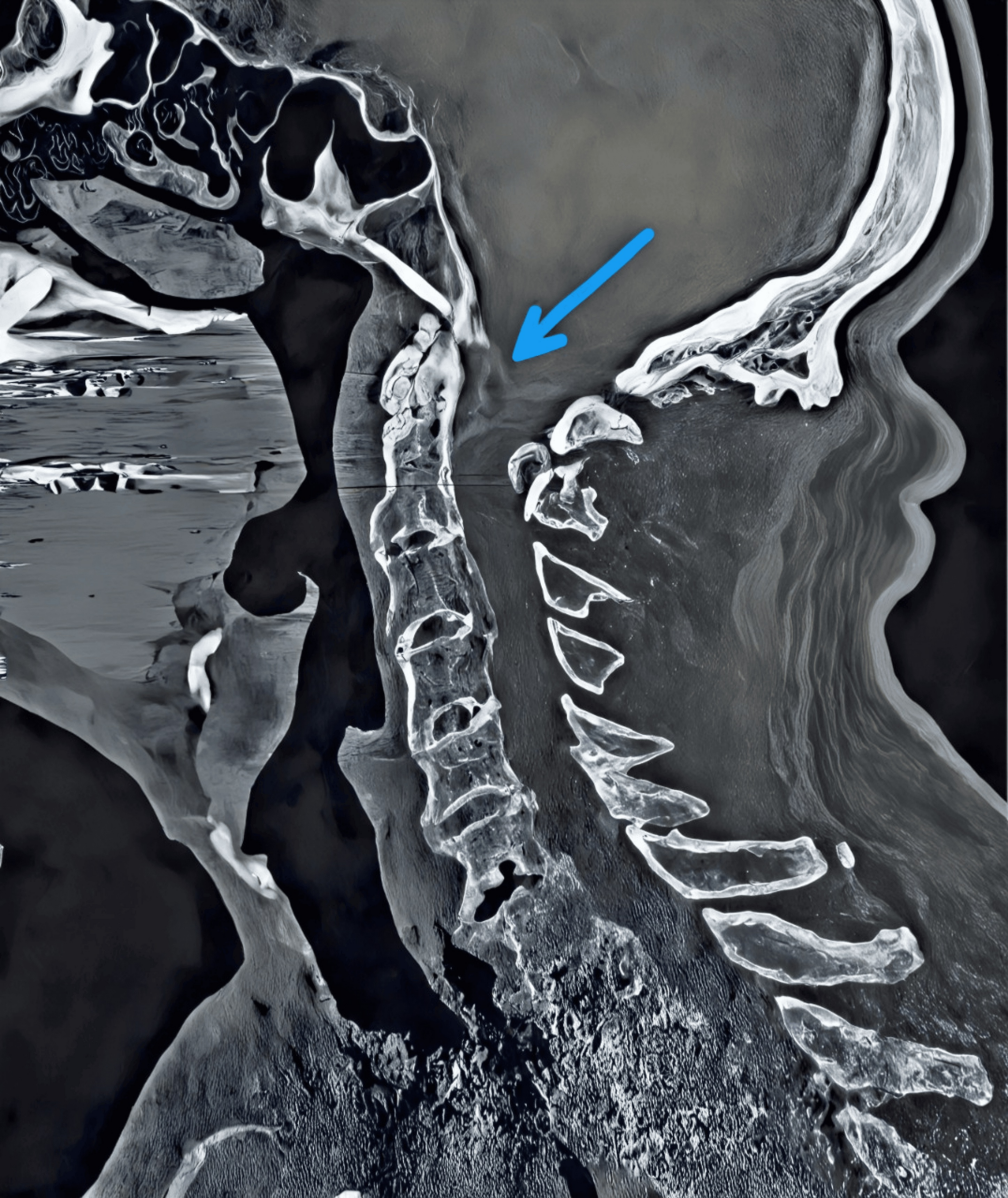
Computed tomography angiography of the cervical spine Midsagittal plane demonstrating the presence of a calcified compressive mass located in the retrodental region.

Magnetic resonance imaging (MRI) of the cervical spine demonstrated significant stenosis at the C1-C2 level (Figure 1) due to a compressive lesion, with fusion from C3 to C5, and signs of spinal cord compression and myelomalacia. Cervical spine CT angiography revealed a mass compressing the anterior spinal cord at the C1-C2 level with associated inflammatory changes (Figure 2).

The clinical presentation, examination findings, and imaging were consistent with advanced cervical myelopathy. Due to the patient’s worsening symptoms and limited response to conservative treatments, including oral corticosteroids and physiotherapy, surgical intervention was deemed necessary. A C1-C2 laminectomy and C1-C3 posterolateral fusion were performed to alleviate the compression.

**Figure 3:**
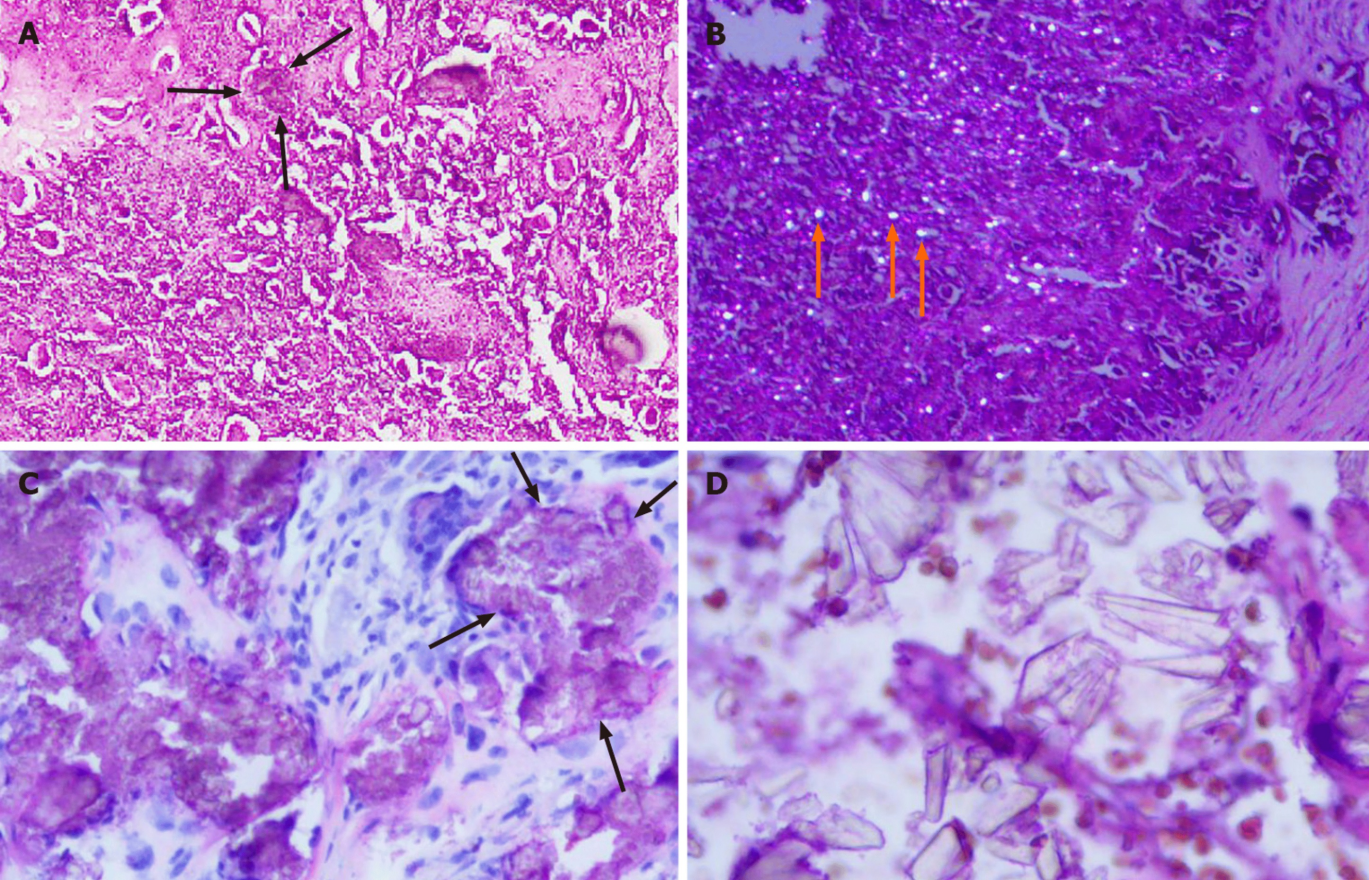
Histopathology A: There are crystal deposits (black arrows) in the fibrous tissue. The crystal deposits are surrounded by foreign body-type giant cells and fibroblasts (hematoxylin-eosin staining, × 100); B: Crystalline material (orange arrows) deposits exhibiting birefringence under polarized light (hematoxylin-eosin staining, × 100; Case 1); C: Nodular clusters of calcium pyrophosphate dihydrate crystals (black arrows) could be seen (hematoxylin-eosin staining, × 200); D: Crystals of different shapes with strong stereoscopic effect (hematoxylin-eosin staining, × 400).

Intraoperatively, deposits of the crystalline material were noted within the ligamentum flavum at the C1-C2 level, as well as involvement of adjacent facet joints. These findings raised suspicion of crystal arthropathy. Pathological analysis of the excised tissue (Figure 3) confirmed the presence of CPPD crystals, indicating pseudogout.

At her six-week follow-up, the patient reported substantial improvement in her left upper extremity strength and sensation, along with marked improvements in her balance. She was able to walk unassisted and had regained sufficient hand dexterity to manage everyday tasks like dressing and typing.

## Discussion

This case highlights a rare and unusual presentation of pseudogout, which is typically more common in larger, weight-bearing joints such as the knees and hips. However, in this instance, it affected the ligamentum flavum and facet joints of the cervical spine, underscoring the importance for spine surgeons to be aware that calcium pyrophosphate crystals can deposit in areas near the spinal cord. These deposits can lead to significant neurological impairment. Therefore, it is crucial to include inflammatory arthropathies like pseudogout in the differential diagnosis when evaluating patients with acute neck pain, especially those presenting with cervical myelopathy or radiculopathy [3].

In this patient, the clinical spectrum of pseudogout was manifested through neck pain, stiffness, and upper extremity weakness. However, CPPD’s presentation is highly variable, and in some cases, neck pain can radiate to the shoulders and occipital region, mimicking conditions like polymyalgia rheumatic [4]. Acute attacks of CPPD can also present with nonspecific symptoms such as fever and headaches, which can resemble conditions like meningitis [4].

The diagnosis of pseudogout in this case was confirmed histologically through pathological examination of the ligamentum flavum, as imaging alone did not provide conclusive evidence of CPPD deposits. Similar cases have been reported in the literature, such as Kobayashi et al.'s case of cervical spine CPPD, where CT scans revealed a high-density area between C5 and C6, suggestive of crystal deposition. Their findings on MRI demonstrated signal changes associated with spinal cord compression, highlighting the usefulness of MRI in detecting CPPD despite its nonspecific radiologic features [3].

In our case, surgical decompression was necessary due to the extent of spinal cord compression. However, the management of CPPD varies, and in cases of mild spinal cord compression with elevated inflammatory markers, conservative treatments such as NSAIDs, corticosteroids, or colchicine may be sufficient. Surgical intervention remains the gold standard for patients with severe myelopathy [5]. However, complications like dural tears should be considered, as calcium deposits can tightly adhere to surrounding structures [5]. Additionally, prolonged CPPD involvement may lead to irreversible spinal cord damage, so early recognition and treatment are critical to preventing long-term neurological deficits. One limitation of this case report is the presence of multiple pathologies in the cervical spine, making it difficult to attribute specific symptoms solely to CPPD. Moreover, with a short follow-up period, it remains unclear whether the patient will experience a recurrence of CPPD in the future.

## Conclusions

This case highlights the importance of recognizing pseudogout (CPPD deposition disease) as a potential cause of cervical spine myelopathy, a condition more commonly associated with larger joints. The involvement of CPPD in the cervical spine can lead to significant neurological deficits, particularly when it affects structures like the ligamentum flavum and facet joints. While MRI and CT imaging may suggest crystal deposition, a definitive diagnosis requires histological confirmation.

Early identification and appropriate management are critical to prevent permanent neurological damage. Conservative treatment may suffice for mild cases, but surgical decompression is necessary for patients with progressive symptoms. Surgeons should anticipate potential complications, such as dural tears, during surgical interventions due to crystal adherence to the dura mater. This case emphasizes the need to consider CPPD in the differential diagnosis of acute neck pain, particularly when myelopathy is present.
